# Diagnostic Algorithm in Patients with Flexion Instability after Cruciate-Retaining Total Knee Arthroplasty: A Case Report

**DOI:** 10.3390/clinpract11030084

**Published:** 2021-09-18

**Authors:** Lukas B. Moser, Ponnaian Prabhakar, Silvan Hess, Michael T. Hirschmann

**Affiliations:** 1 Department of Orthopaedic Surgery and Traumatology, Kantonsspital Baselland (Bruderholz, Liestal, Laufen), Bruderholz, CH-4101 Basel, Switzerland; silvanhess@msn.com (S.H.); michael.hirschmann@unibas.ch (M.T.H.); 2 Research Group Michael T. Hirschmann, Regenerative Medicine & Biomechanics, Department of Clinical Research, University of Basel, CH-4001 Basel, Switzerland; 3 Department of Orthopaedics, Trauma and Arthroplasty, Care Superspeciality Hospitals, Nampally, Hyderabad 500 001, India; drprabhakar1@gmail.com

**Keywords:** cruciate retaining, total knee arthroplasty, total knee replacement, posterior cruciate ligament, PCL

## Abstract

A posterior flexion instability due to insufficiency of the posterior cruciate ligament (PCL) in cruciate retaining (CR) total knee arthroplasty (TKA) is an important but underdiagnosed problem. We hereby suggest a diagnostic algorithm, as demonstrated by a case report of a male patient suffering from anterior knee pain and instability after CR TKA. Clinical examination was followed by standard anterior–posterior and lateral radiographs. Stress radiographs in 30° and 90° posterior drawer position enabled a dynamic examination of the instability. SPECT/CT was used to determine the TKA component position in all planes and investigate bone tracer uptake (BTU) patterns. At revision surgery, an absent PCL after CR TKA was noted and a semi-constrained TKA was implanted.

## 1. Introduction

Although total knee arthroplasty (TKA) is an effective treatment for end-stage osteoarthritis (OA), up to 30% of the patients are not satisfied and pain-free [1]. Early or late infection, aseptic loosening, malposition of the TKA, patellofemoral problems, and instability are considered to be the most common causes for unhappy knees after TKA [2].

Flexion instability, together with instability in extension and genu recurvatum, is responsible for 10–22% of revision surgeries [3]. In cruciate retaining (CR) TKA, the posterior cruciate ligament (PCL) limits posterior tibial translation and, hence, should not be sacrificed [4]. Iatrogenic harm at TKA or secondary PCL insufficiency, due to a tight flexion gap or secondary degenerative changes, lead to an increased posterior translation and flexion instability [1]. 

However, a careful review of orthopedic literature reveals that reports on PCL insufficiency in patients after primary CR TKA are rare and a diagnostic algorithm does not yet exist. Establishment of the correct diagnosis is challenging as clinical examination and standard radiographs often do not reveal the problem of a PCL insufficiency in CR TKA. Stress radiographs help to identify the problem and quantify the amount of posterior translation. In recent years, SPECT/CT has been increasingly used by knee surgeons for the assessment of patients with unhappy TKA [5]. The use of SPECT/CT is especially beneficial in the context of posterior instability in CR TKA. It allows for visualization of the biological consequences of an increased posterior tibial translation (e.g., increased patellar uptake) and 3D-reconstructed CT scans provide information on femoral and tibial component position.

We hereby suggest a diagnostic algorithm based on a case report of a male patient with suspected PCL insufficiency suffering from anterior knee pain and instability after CR TKA.

## 2. Case Report

**Figure 1:**
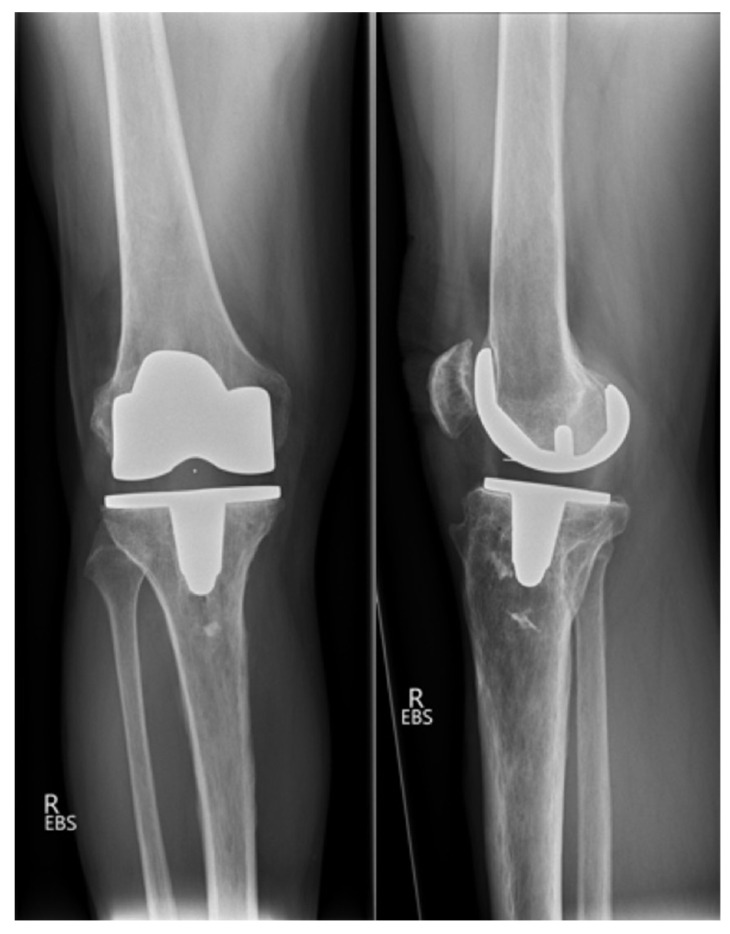
Anteroposterior and lateral non-weight bearing radiographs of the right knee after primary posterior cruciate retaining TKA.

A 62-year-old male patient presented with a painful right knee eight months after CR TKA. The patient described a subjective feeling of instability, especially when walking downstairs or on uneven ground. Pain could also be provoked by prolonged sitting with a flexed knee. The patient noted that the character of pain was different than prior to TKA. Cycling was possible without any limitations. At clinical examination, there was mild local hyperthermia and peripatellar pain on palpation. Internal and external rotation was unremarkable; in flexion, a lift-off of the lateral femoral condyle was observed. Active range of motion (flexion/extension) was 130°/0°/5°. Posterior drawer testing showed an increased posterior translation in comparison to the contralateral knee. Anterior–posterior and lateral radiographs of the knee were unremarkable and showed a well-fixed prosthesis with no evidence of loosening (Figure 1). 

**Figure 2:**
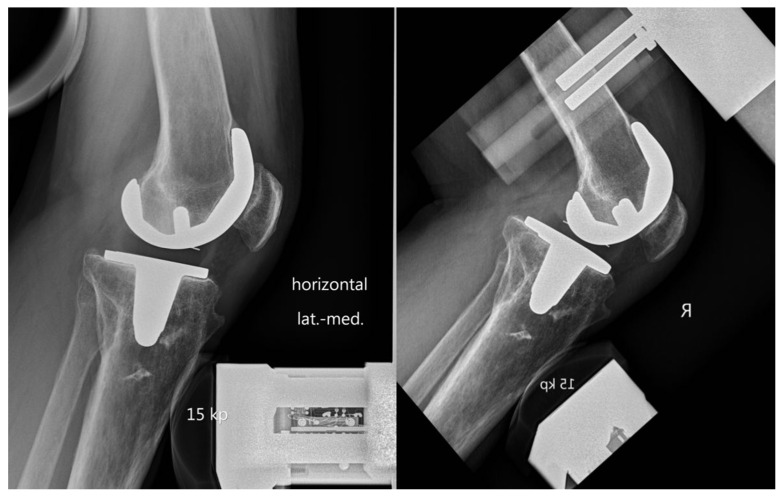
Stress radiographs showed increased posterior translation in posterior drawer position in 90° flexion compared to 30° flexion.

Flexion instability due to PCL insufficiency was suspected and, thus, stress radiographs in 30° and 90° flexion were performed. There were no signs of lateral or medial lift-off in extension, and anterior tibial translation presented normal in flexion and extension. Posterior drawer position in 90° flexion confirmed clinical diagnosis of PCL insufficiency and showed a 13-mm increased posterior translation of the tibia (Figure 2). It is important to note here that primary TKA was carried out in a different clinic without any knowledge of PCL damage at the time of the operation. 

**Figure 3:**
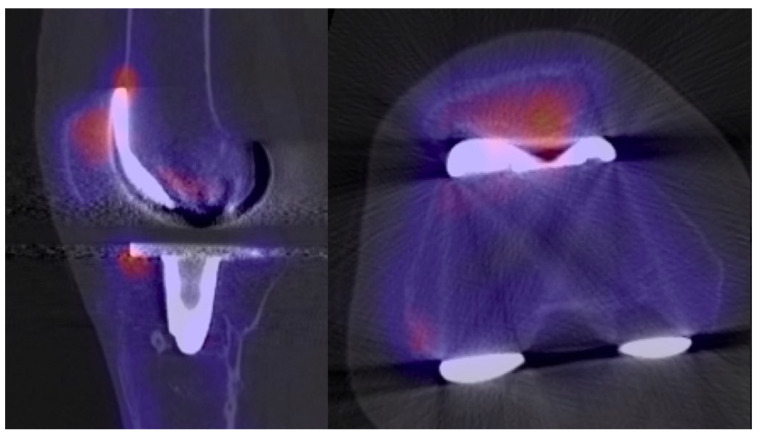
Increased bone tracer uptake on the patella in sagittal and axial plane indicating increased patella loading due to posterior instability.

As part of the routine, a diagnostic algorithm in our clinic for unhappy patients after TKA, SPECT/CT using 99 mTc-hydroxymethylene diphosphate (HDP) as bone tracer was performed. It revealed an increased bone tracer uptake (BTU) in the entire patella (Figure 3) which is a typical pattern for posterior knee instability and consecutively increased patellar loading. There was also an increased uptake detected at the medial tibial plateau.

**Figure 4:**
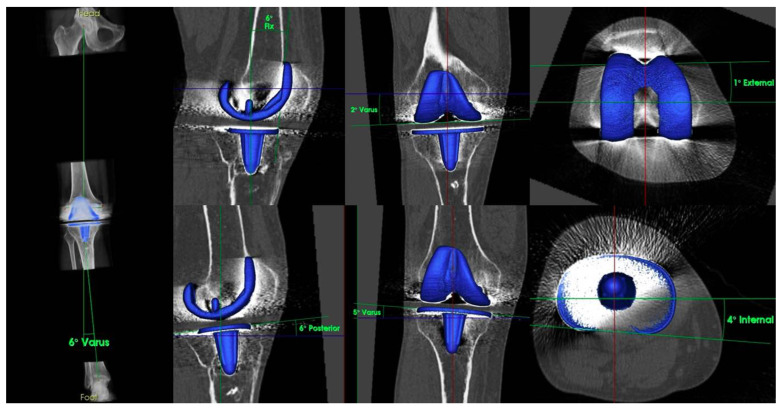
TKA component position in 3D reconstructed CT scans.

A TKA component position was evaluated in coronal, axial, and sagittal plane using 3D-reconstructed CT scans (Figure 4). Mechanical axis was in 6° varus and the coronal tibial component showed a malposition of 5° varus, which explains the increased BTU at the medial tibia.

**Figure 5:**
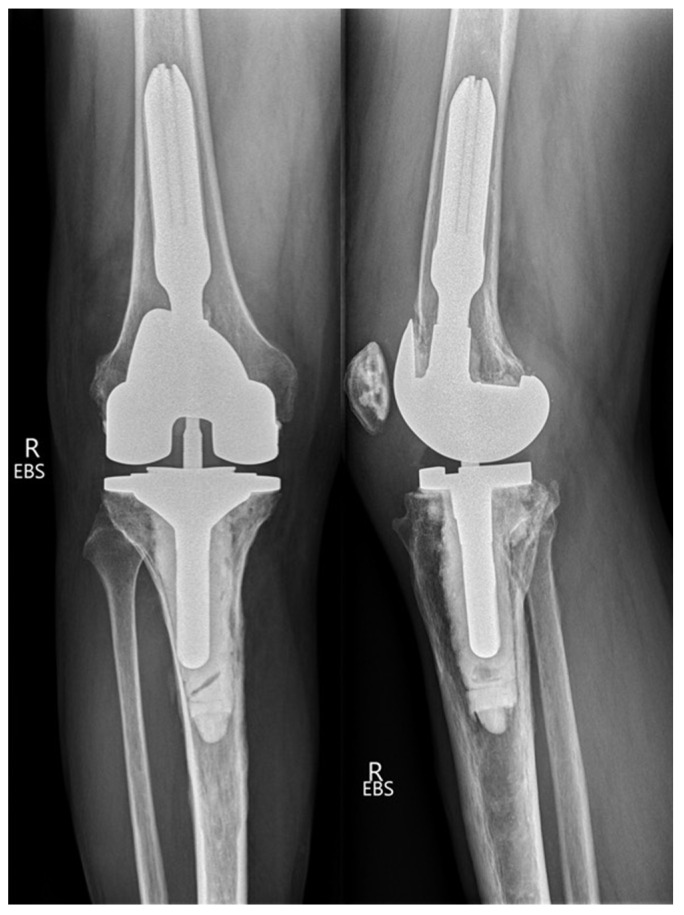
Anteroposterior and lateral non-weight bearing radiographs of the right knee after revision surgery with a semi-constrained TKA.

Based on the symptomatic posterior flexion instability and the malposition of the tibial component, the decision was made to perform revision surgery. At revision surgery, the PCL was found to be torn and the knee prosthesis was changed to a semi-constrained TKA (Figure 5). At follow-up of 36 months after revision TKA, the patient was pain-free, and had a full range of motion and a stable knee.

## 3. Discussion

This case report highlights the importance of an intact PCL in patients after CR TKA and offers a comprehensive diagnostic algorithm including clinical examination and different imaging modalities. Posterior flexion instability of the knee appears to be an underdiagnosed problem in patients with ongoing pain after TKA. One reason for this might be that fact that many studies relied on conventional radiographs that do not project the posterior translation accurately.

Montgomery et al. reported three cases of a late PCL rupture in a series of 150 PCL-retaining prosthesis [6]. They suspected an insufficiency because of clinical and radiological findings and performed revision arthroplasty in each case. Pagnano et al. compiled a case series of 25 painful primary CR TKA suffering from flexion instability [7]. All patients were revised, 22 to a posterior stabilized (PS) implant and 3 underwent exchange of the polyethylene inlay. Improved knee society scores (KSS) after revision surgery led the authors to conclude that a revision with a PS design is an effective treatment for flexion instability after CR TKA. Waslewski et al. identified 13 patients with 16 PCL-retaining TKAs suffering from an instability due to early PCL deficiency [8]. Patients presented to his clinic with a triad of persistent swelling, anterior knee pain, and of instability episodes. However, diagnosis only included physical examination and conventional X-rays. After showing no improvement on pain or instability after physical therapy and usage of PCL brace, six patients underwent revision surgery to a PS TKA. They consequently reported an improvement of pain and instability. The authors believe that early PCL deficiency is underreported and should be considered in patients with anterior knee pain with normal radiographs.

Stress radiographs are able to overcome limitations of standard ap and lateral radiographs. This finding is very well demonstrated in the present case report. Although clinical examination showed an increased posterior translation, standard radiographs were normal. Ultimately, the posterior drawer position in ongoing flexion (90°) revealed increased posterior translation (13 mm). Although some authors have reported threshold values for stress radiographs after CR TKA [9], recommendations for diagnosing posterior instability have not yet been established.

Reasons for PCL insufficiency in CR designs are manifold. Iatrogenic damage can occur directly during primary TKA by harming or even resection of the PCL [3]. Late PCL ruptures are usually associated with weakness due to age-related degenerative changes and/or traumatic injuries such as knee dislocation [6]. However, the implantation technique, and consequently the position of the femoral and tibial component, can influence the PCL indirectly. An excessive tibial slope [3] and/or a too-tight flexion gap can harm the PCL and lead to early PCL failure [10]. Evaluating the component position is certainly a crucial part in the diagnostic algorithm of patients with unhappy TKA. A thorough evaluation should include all planes: coronal, sagittal, and axial, which is clearly not possible on radiographs. 3D-reconstructed CT scans are regarded as the gold standard in diagnosing the component position in TKA and, therefore, should be included in the diagnostic algorithm of patients with posterior instability after TKA [5]. In recent decades, SPECT/CT has evolved rapidly. It is widely used in nuclear medicine departments worldwide and as part of the diagnostic routine including many diseases of the oncological (e.g., thyroid cancer, neuroendocrine neoplasms, bone metastasis) and non-oncological (e.g., infection, lung disorders, cardiology) spectrum [11]. In the context of knee surgery, SPECT/CT offers another benefit in assessing patients with posterior instability by combining the conventional CT with SPECT. Whereas CT component delivers information on alignment, the SPECT component gives information on bone physiology. Several studies have shown that increased bone tracer uptake (BTU) reflects areas of increased in-vivo loading and BTU patterns for different problems have been detected [12,13,14,15,16]. Knees suffering from posterior instability cause the tibia to shift backwards due to PCL insufficiency which increases the pressure on the patella and patients report of knee pain. Combining the information of alignment of the components with BTU reveals whether the anterior knee pain was due to an increased tibial slope or other reasons (e.g., malrotation of the femoral component). The present case demonstrated the benefits of SPECT/CT very well. Increased BTU was found at the patella, but the tibial slope was normal (6°) and femoral component was not malrotated (1° of external rotation). However, increased BTU at the medial tibia was found as a consequence of coronal malposition of the tibial component in 5° varus. In terms of costing, Van den Wyngaert et al. investigated the cost-effectiveness of SPECT/CT in painful total knee arthroplasty. The authors listed the Medicare national payment amount according to the American Medical Association. Accordingly, a SPECT/CT scan costs about USD 239.38 per scan, a regular CT scan is about USD 115.20, and a metal artifact reduction sequence (MARS)-MRI costs USD 646.72. Interestingly, the authors also performed a cost-utility analysis in order to estimate the incremental cost per quality-adjusted life years gained between bone SPECT/CT and CT or MRI. They have shown that, for every 1000 TKA patients, SPECT/CT is expected to lead to 3 years of cost savings up to USD 622.6 per patient per year versus CT and USD 574.5 per patient per year versus MARS-MRI. This study shows that costs of SPECT/CT might be higher than regular CTs cans, but its benefit in diagnosing the patient more accurately leads to increased costs for the healthcare in the long term.

**Figure 6:**
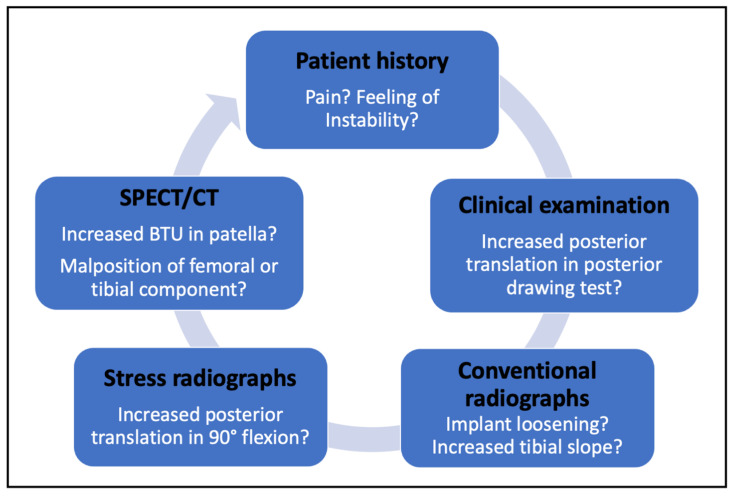
This figure highlights the diagnostic workup for patients with flexion instability after CR TKA proposed by the present case report.

This case report illustrates that SPECT/CT is a very helpful imaging modality and is not necessarily restricted for application in patients with suspected PCL insufficiency. In fact, all patients with knee pain following TKA should receive SPECT/CT as part of the diagnostic workup. Likewise, stress radiographs should be done in all patients suffering from patella femoral pain in order to detect potential PCL insufficiency after TKA. The combination of anterior knee pain, posterior instability, and varus malposition of the tibial component, evaluated by using the present diagnostic algorithm, finally led to the decision of revision surgery (Figure 6).

## 4. Conclusions

In the case of flexion instability in CR TKA, a thorough examination using different imaging modalities is necessary. A comprehensive diagnostic algorithm should include the patient’s history, a thorough clinical examination, conventional and stress radiographs, and SPECT/CT including measurements of BTU and TKA position.
